# Insulin and IGF1 signalling pathways in human astrocytes *in vitro* and *in vivo*; characterisation, subcellular localisation and modulation of the receptors

**DOI:** 10.1186/s13041-015-0138-6

**Published:** 2015-08-22

**Authors:** Claire J. Garwood, Laura E. Ratcliffe, Sarah V. Morgan, Julie E. Simpson, Helen Owens, Irina Vazquez-Villaseñor, Paul R. Heath, Ignacio A. Romero, Paul G. Ince, Stephen B. Wharton

**Affiliations:** Sheffield Institute for Translational Neuroscience, Department of Neuroscience, The University of Sheffield, 385a Glossop Road, Sheffield, S10 2HQ UK; Biomedical Research Network, The Open University, Walton Hall, Milton Keynes, MK7 6AA UK

## Abstract

**Background:**

The insulin/IGF1 signalling (IIS) pathways are involved in longevity regulation and are dysregulated in neurons in Alzheimer’s disease (AD). We previously showed downregulation in IIS gene expression in astrocytes with AD-neuropathology progression, but IIS in astrocytes remains poorly understood. We therefore examined the IIS pathway in human astrocytes and developed models to reduce IIS at the level of the insulin or the IGF1 receptor (IGF1R).

**Results:**

We determined IIS was present and functional in human astrocytes by immunoblotting and showed astrocytes express the insulin receptor (IR)-B isoform of *Ir*. Immunocytochemistry and cell fractionation followed by western blotting revealed the phosphorylation status of insulin receptor substrate (IRS1) affects its subcellular localisation. To validate IRS1 expression patterns observed in culture, expression of key pathway components was assessed on post-mortem AD and control tissue using immunohistochemistry. Insulin signalling was impaired in cultured astrocytes by treatment with insulin + fructose and resulted in decreased IR and Akt phosphorylation (pAkt S473). A monoclonal antibody against IGF1R (MAB391) induced degradation of IGF1R receptor with an associated decrease in downstream pAkt S473. Neither treatment affected cell growth or viability as measured by MTT and Cyquant® assays or GFAP immunoreactivity.

**Discussion:**

IIS is functional in astrocytes. IR-B is expressed in astrocytes which differs from the pattern in neurons, and may be important in differential susceptibility of astrocytes and neurons to insulin resistance. The variable presence of IRS1 in the nucleus, dependent on phosphorylation pattern, suggests the function of signalling molecules is not confined to cytoplasmic cascades. Down-regulation of IR and IGF1R, achieved by insulin + fructose and monoclonal antibody treatments, results in decreased downstream signalling, though the lack of effect on viability suggests that astrocytes can compensate for changes in single pathways. Changes in signalling in astrocytes, as well as in neurons, may be important in ageing and neurodegeneration.

**Electronic supplementary material:**

The online version of this article (doi:10.1186/s13041-015-0138-6) contains supplementary material, which is available to authorized users.

## Introduction

Insulin and insulin-like growth factor (IGF) signal primarily through the phosphatidylinositol-4,5-bisphosphate 3-kinase (PI3K)/Akt and Ras/Mitogen activated protein kinase (MAPK) pathways to affect multiple cellular functions including cell growth, cell survival and cellular metabolism [1]. These complex signalling pathways are increasingly implicated in the pathogenesis of Alzheimer’s disease (AD) and other neurodegenerative diseases including Parkinson’s disease and motor neurone disease [2–4]. An insulin resistant state is evident in the brain early in AD progression [5, 6], and a number of epidemiological studies have identified Type-2 diabetes (T2D), in which an insulin resistant state exists, as a risk factor for developing AD [7–9]. These pathways have also been implicated as regulators of longevity [1] and may be important in brain ageing and its interaction with neurodegeneration.

Astrocytes outnumber neurons in human brain [10] and are responsible for complex and essential brain functions. They form part of the tripartite synapse, integrating and processing synaptic information [11], produce and release neurotrophic factors to promote neuronal survival; and regulate cerebral metabolic trafficking between neurons and intracerebral blood vessels [12–14]. In addition astrocytes play a key role in restoring brain homeostasis in brain injuries and neurodegenerative disease [15, 16] although their role in ageing and neurodegeneration is complex and not fully understood. The prototypical astrocyte response is gliosis [14] although astrocyte atrophy has also been observed in AD mouse models [17] suggesting loss of function as well as altered function can occur.

We have previously shown that astrocyte hypertrophy and injury occur early on in the progression of AD [18, 19] in an ageing cohort and that astrocytic calcium signalling is disrupted as Alzheimer’s neuropathology progresses [20, 21]. Microarray analysis of the transcriptome of astrocytes isolated from temporal neocortex by laser capture microdissection found that the insulin/IGF1 signalling (IIS) pathways, together with their downstream targets, MAPK and PI3K/Akt, are downregulated as Alzheimer-type pathology progresses [21]. Altered signalling may lead to loss and/or altered function in ageing and disease and may also indicate an altered interaction with the neurovascular unit.

**Fig. 1:**
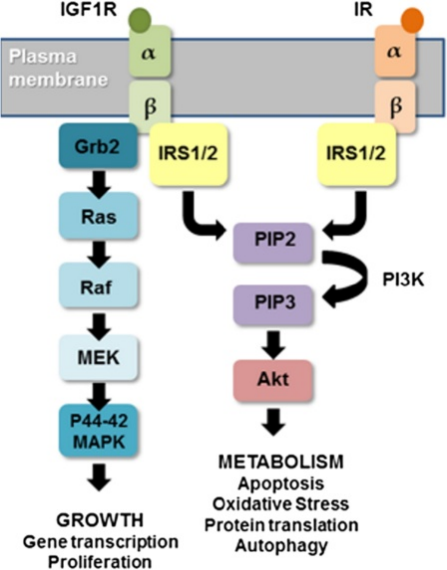
Schematic of the insulin/IGF1R signalling pathway. A simplified representation of the insulin/IGF1 signalling pathway depicting the downstream activation of Akt and p44-42 MAPK through binding of insulin or IGF1 to their respective receptors. Furthermore insulin can bind to IGF1R and IGF1 to IR and IR α-subunits/ β-subunits can form heterodimers with IGF1R α-subunits /β-subunits adding further complexity at the level of the receptors. There are also numerous downstream feedback loops within this and other signalling pathways which act to regulate signalling through this pathway at multiple levels

Insulin acts through the insulin receptor (IR), a heterotetrameric receptor tyrosine kinase (RTK) composed of two extracellular alpha sub-units, which have ligand-binding activity, and two transmembrane beta subunits that possess tyrosine kinase activity. Similarly the IGF1 receptor (IGF1R) is a RTK with a transmembrane complex which is identical to IR. Both receptors show a high degree of homology; 84 % in the tyrosine kinase domain and 45–65 % in the ligand binding domain [22]. As such both ligands can bind and activate both RTKs and therefore the interaction of these two pathways is important. Binding of insulin or IGF1 triggers autophosphorylation of tyrosine residues within the beta subunit which, in turn, leads to the recruitment of adaptor proteins, namely insulin receptor substrate (IRS)1 and IRS2, as well as Gab1 and Dos [23]. A schematic representation of IIS is shown in Fig. 1.

Previous experimental investigations of the role of IIS in AD progression have focused on mouse models. However, modelling these systems in mice may not fully recapitulate the nuances of insulin signalling in humans as it has recently been demonstrated *in vitro* that there are clear distinctions in rodent and human cell responses to insulin concentration [24]. Furthermore the majority of studies to date have focused on altered signalling and insulin resistance in neurons.

We have characterised the insulin and IGF1 signalling pathways in human primary astrocytes and have developed models in which insulin or IGF1 signalling are impaired in human astrocytes in order to investigate the functional implications of impaired insulin signalling in astrocytes. The use of human astrocytes is important as there are clear differences in astrocytes complexity between rodents and humans, with human astrocytes being larger and structurally more complex and more diverse, than those of rodents [25]. We show that the insulin/IGF1 signalling pathways are functional in human astrocytes and that human astrocytes express the IR-B isoform of the insulin receptor. We demonstrate that IRS1 localisation is dependent on its phosphorylation state and report the development of models for the modification of these pathways; using a combined insulin-fructose treatment protocol we specifically impair insulin signalling in these cells, and through the use of an IGF1R monoclonal antibody we impair IGF1 signalling through this pathway.

## Results

### Characterisation of human primary astrocytes

Human astrocytes from Sciencell and from temporal lobe resections were cultured in two defined media to assess growth rate, morphology and differentiation-marker expression. Astrocytes cultured in isolation and in the presence of serum showed a heterogeneous morphology, with variations in both the size and extent of processes as well as in overall cell size (Additional file 1: Figure S1a). They expressed the intermediate filament proteins vimentin and glial fibrillary acidic protein (GFAP), and the cell surface glycoprotein CD44 (Additional file 1: Figure S1, Sciencell astrocytes), which is consistent with an astrocyte phenotype. All astrocytes were cultured in 2 different media, a specific commercial astrocyte media from Sciencell Research Laboratories and a defined media for culturing human primary astrocytes [26]. The cells grew more rapidly in Sciencell media compared to F10:MEMα media (Additional file 2: Figure S2a, Sciencell astrocytes). In contrast, the expression of IRβ was lower in the Sciencell media (Additional file 2: Figure S2b, Sciencell astrocytes) and therefore all subsequent experiments were performed in F10:MEMα media. Unless specifically stated the results below relate to experiments conducted on Sciencell astrocytes.

### Human astrocytes predominantly express IR-B

**Fig. 2:**
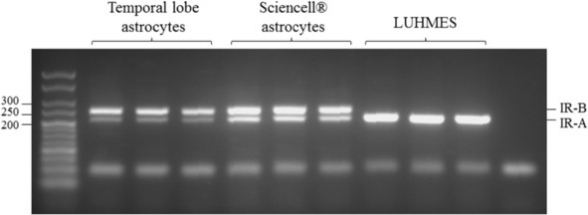
Insulin receptor isoform expression in human astrocytes and LUHMES. Representative gel from RT-PCR assesses the expression of insulin receptor isoforms in astrocytes derived from temporal lobe resections (adult), Sciencell astrocytes (fetal), and differentiated LUHMES using primers spanning exon 11 of the *Ir* (present only in IR-B)

We sought to determine whether the IR isoform expressed by human astrocytes was different from that in a human neuronal cell line. The isoforms differ by the inclusion (IR-B) or exclusion (IR-A) of exon 11 which encodes a 12 amino acid region in the C-terminus of the receptor. As shown in Fig. 2 the IR isoform profile differs in human astrocytes to neurons (LUHMES) with IR-B predominating in astrocytes whilst neurons expressed exclusively IR-A. We also compared IR isoform expression in our 2 different sources of human astrocytes (fetal and adult) and found the expression pattern to be similar (a predominance of IR-B over IR-A). To our knowledge this is the first paper confirming IR isoform expression in adult human astrocytes.

### The insulin/IGF1 signalling pathway is present and functional in human astrocytes

The insulin and IGF1 signalling pathways in human astrocytes were characterised when cells were cultured in the presence (complete medium) or absence of serum (serum-deprived medium) for 24 h. Astrocytes were additionally supplemented with either 1 μM recombinant human insulin or 13.2 nM human recombinant IGF1 for 2 h to determine whether insulin/IGF1 signalling in complete medium resulted in full activation of the pathway.

**Fig. 3:**
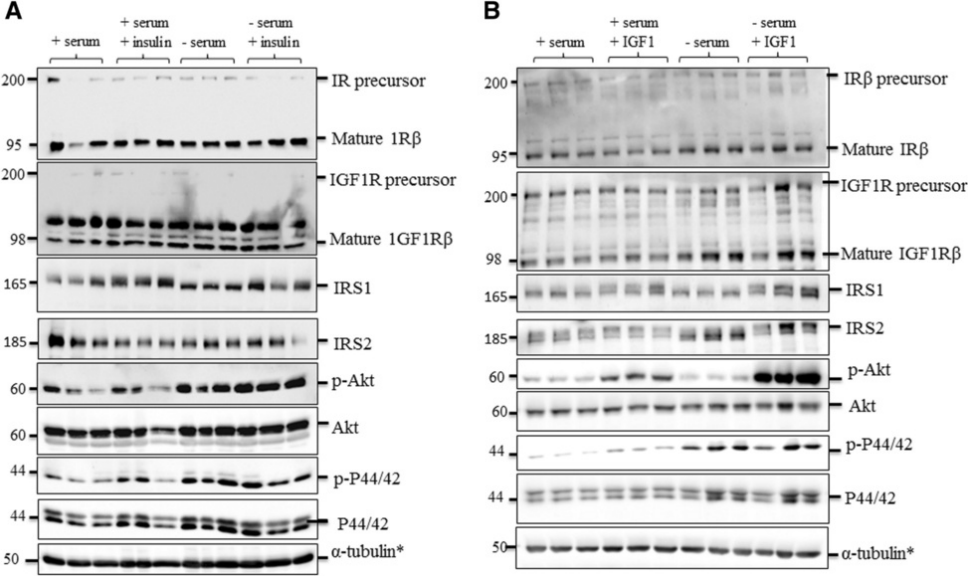
Insulin/IGF1 pathway characterisation in astrocytes. Immunoblots of astrocytes cultured in either serum containing or serum deprived media with additional supplementation from either **a** recombinant human 1 μM insulin or **b** 11.2nM recombinant human IGF1. Respresentative images from blots probed with antibodies against IRβ, IGF1Rβ, IRS1, IRS2, pAkt, Total Akt, p44/42 MAPK are shown. *α-tubulin was used as a loading control for blots and a representative loading control is shown. Molecular weight markers are indicated (kDa)

Cultured human astrocytes expressed the receptors IR and IGF1R, the adaptor proteins IRS1 and IRS2 and the downstream signalling kinases Akt and p44/42 MAPK (ERK1/2) in both the commercially obtained Sciencell astrocytes and astrocytes derived from temporal lobe resections (see Fig. 1 for schematic representation of the insulin/IGF1 signalling pathway). There was no impact of serum deprivation or insulin/IGF1 supplementation on either the pre- or mature receptors (Fig. 3 and Additional file 3: Figure S3). Amounts of the adaptor protein, IRS1, remained consistent although the detected molecular weight increased in the presence of serum or when astrocytes were supplemented with recombinant insulin or IGF1 (Fig. 3 and Additional file 3: Figure S3), indicating a change in the phosphorylation status of the protein; the complex regulation of IIS is achieved through phosphorylation of IRS1 on tyrosine and serine residues. A shift in IRS2 was also observed by immunoblotting in response to stimulation with ligand, indicating that insulin/IGF signalling is mediated by both downstream adaptors proteins, IRS1 and IRS2 (Fig. 3).

Binding of the receptor and subsequent phosphorylation of IRS1/2 results in phosphorylation and activation of downstream targets including Akt. Phosphorylation of Akt at serine residue 473 (pAkt S473) results in full activation of the kinase [27, 28]. Immunoblotting for pAkt s473 showed that addition of insulin or IGF1 to astrocytes cultured in complete medium had little effect on pAkt S473 (relative to total Akt) (Fig. 3), suggesting that the growth factors present in serum fully activate the pathway. However, in serum deprived astrocytes a large increase in pAkt S473 was observed when either insulin or IGF1 were added to the media, indicating increased Akt activity (Fig. 3). In astrocytes derived from temporal lobe resections serum starvation completely abrogated Akt activation and although supplementation with insulin resulted in an increase in pAkt S473 it did not reach the levels seen in cells cultured with serum (Additional file 3: Figure S3) suggesting that basal Akt activity and its regulation differs between these astrocytes. Taken together these date demonstrate that the IIS pathway is present and functional in these cells.

### The localisation of IRS1 in astrocytes is dependent on phosphorylation state

**Fig. 4:**
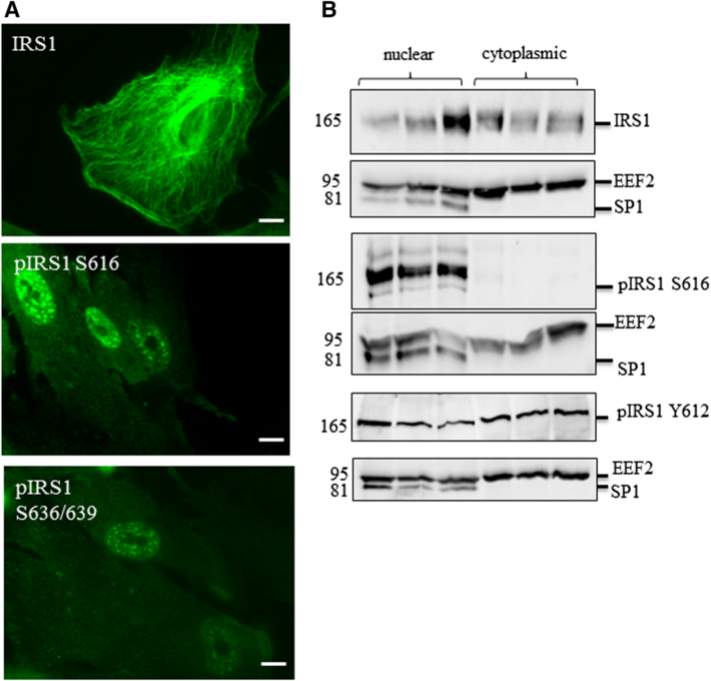
Localisation of signalling components in human astrocytes. **a** Immunocytochemistry demonstrates nuclear localisation of IRS1 phosphorylated at serine 616 (S616) and S636/639 but not total IRS1 **b** Representative immunoblots of fractionated cell lysates showing the localisation of total IRS1 and IRS1 phosphorylated at S616 and tyrosine 612 (Y612) by immunoblot. Loading control for cytosplasmic fraction are EEF2 and for nuclear fraction is SP1. Molecular weight markers are indicated (kDa)

In addition to blotting, immunocytochemistry was performed to determine the cellular localisation of the IIS pathway components. IRS1 was localised in both the cytoplasm and the nucleus in astrocytes cultured in complete media. This was confirmed by sub-cellular fractionation followed by immunoblot analysis of IRS1 in human astrocytes (Fig. 4a, b), using eukaryotic elongation factor (EEF2) as a marker for the cytoplasmic fraction and specificity protein 1 (SP1) as a marker for the nuclear fraction. However, IRS1 phosphorylated at S616 was predominantly localised in the nucleus as shown by both immunofluorescence and immunoblotting (Fig. 4a, b). We additionally looked at IRS1 when phosphorylated at S636/639 using immunofluorescence (Fig. 4a), which was also nuclear in localisation. Since tyrosine phosphorylation of IRS1 is associated with activation of IIS [29] we also investigated the localisation of IRS1 when phosphorylated at tyrosine 612 (Y612). We experienced difficulties in obtaining specific staining using immunofluorescence but analysis by fractionation and immunoblot analysis suggests that pIRS1 Y612 is localised to both the nucleus and cytoplasm (Fig. 4b).

### Confirmation of IRS1 localisation in human tissue

**Fig. 5:**
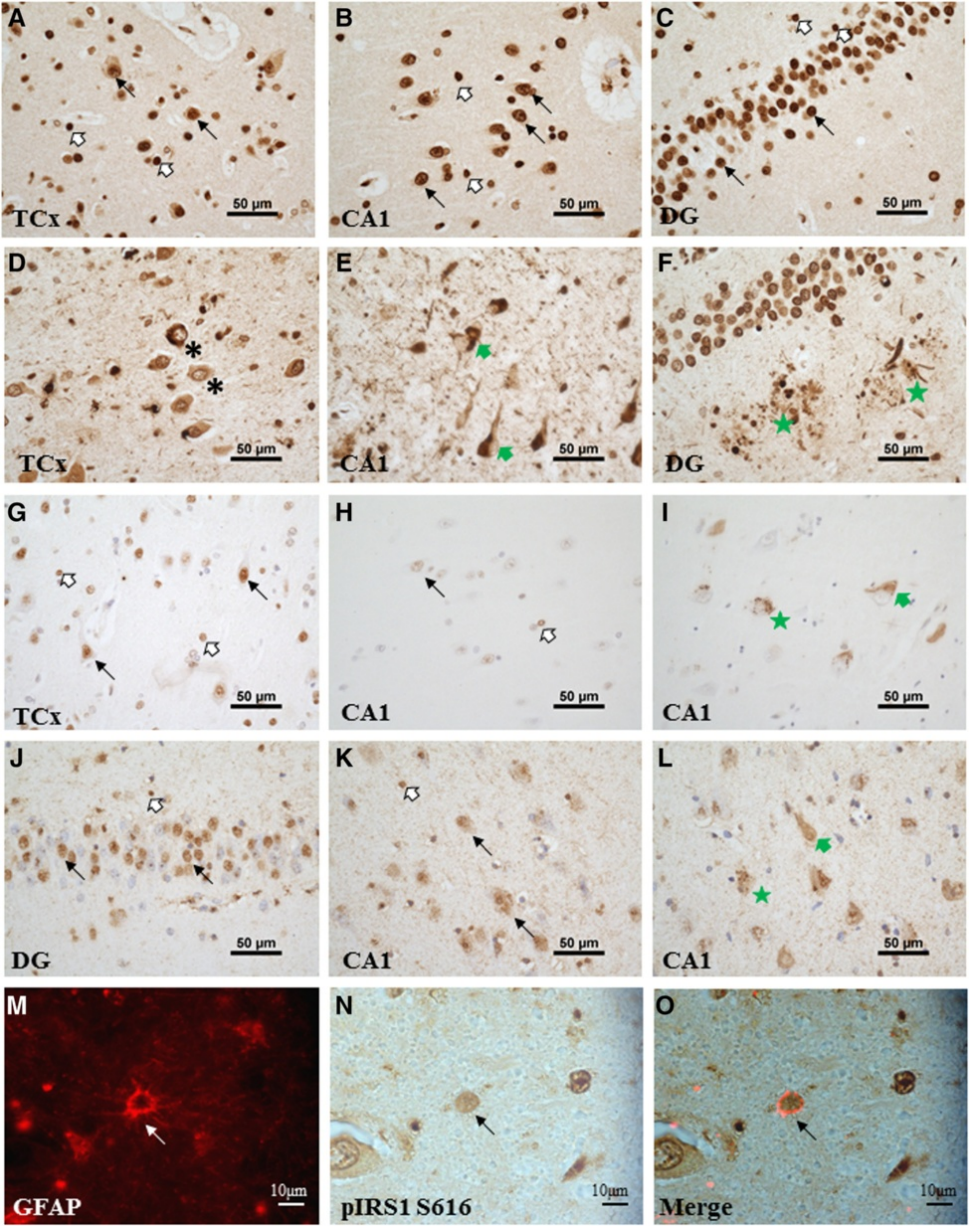
Immunohistochemistry showing the localisation of phospho-IRS1 in human brain (control and Alzheimer’s disease cases [*n* = 3]). Each panel is a representative figure from either 3 control or 3 AD cases. **a**-**f** pIRS1 S616 labelled temporal cortex (TCx) and hippocampus (CA1 and dentate gyrus [DG]). Positive neuronal nuclei (*black arrows*) and small cells (*white arrows*) are evident in control brain (**a**-**c**). In AD brain (**d**-**f**) a large proportion of nuclei are negative (*black asterix*) and neurofibrillary tangles (NFTs) (*green arrows*) and neuritic plaques (*green stars*) are immunopositive for pIRS1 S616. **g**-**i** pIRS1 S636/639 stained TCx and CA1, a similar pattern of staining to pIRS1 S616 is evident in control TCx (**g**) although there is little nuclear immunoreactivity in control CA1 (**h**) and there are immunopositive NFTS (*green arrow*) and dot like structures resembling granulovacular degeneration (GVD) (*green star*) in AD CA1 region (**i**). **j**-**l** pIRS1 Y612 staining was predominantly nuclear in control DG (**j**) (neuronal = *black arrow*, small cells = *white arrow*) and was both cytoplasmic and nuclear in control CA1. In AD brain (**l**) pIRS1 Y612 labelled NFTS (*green arrow*) and GVD (*green arrow*). **m**-**o** demonstrates colocalisation of GFAP-positive astrocytes (*red*) with pIRS1 S616 (*brown*) (CA1 region, AD brain)

To demonstrate the relevance of these findings in culture to the *in vivo* situation, we examined expression of IRS1 in human post-mortem brain tissue from the Sheffield Brain Tissue Bank. Temporal cortex and hippocampal regions from control and AD brains were stained for pIRS1 S616, pIRS1 S636/639 and pIRS1 Y612 (Fig. 5). In control brain pIRS1 S616 labelled the nucleus of pyramidal neurons and small cells with some faint cytoplasmic staining present, this pattern of staining was the same for both the temporal cortex and the hippocampus (Fig. 5a-c). In the temporal cortex of AD cases the nucleus of neurons was pIRS1 S616 negative (Fig. 5d) and there was clear staining of neurofibrillary tangles (NFTS) and neuropil threads (Fig. 5e). In the CA1 region of the hippocampus both positive and negative pIRS1 S616 nuclei were detected, with negative nuclei more apparent in those neurons that had cytoplasmic staining associated with tangles. There were also dot like structures in vacuoles resembling granulovacular degeneration (GVD) and evidence of immunopositive neuritic plaques in the dentate gyrus (Fig. 5f). A similar pattern of staining was observed with pIRS1 S636/639; in control brain IRS1 pS636/639 was predominantly nuclear, and was present in both neurons and small cells (Fig. 5g-h), in AD nuclei were predominantly negative, with this being particularly apparent in neurons which had evidence of cytoplasmic staining associated with tangles, and tau pathology including NFTs and GVD were pIRS1 S636/639 positive (Fig. 5i). pIRS1 Y612 was both nuclear and cytoplasmic in localisation (Fig. 5k) in control CA1 with a proportion, but not all, nuclei in the dentate gyrus being pIRS1 Y612 postive (Fig. 5j). In AD brain pIRS1 Y612 was less nuclear overall with NFTS and GVD both staining (Fig. 5l). In both control and AD brain there was also a punctate granular stain present throughout the neuropil which may indicate labelling of synapses by pIRS1 Y612 (Fig. 5j-l). We also performed dual labelling immunohistochemistry to confirm that astrocytes were positive for pIRS1 S616 (Fig. 5m-o). These results support our *in vitro* work and suggest there may be mislocalisation of IRS1 in neurons in association with tau-pathology in AD.

### Insulin signalling can be impaired by insulin/fructose treatment

**Fig. 6:**
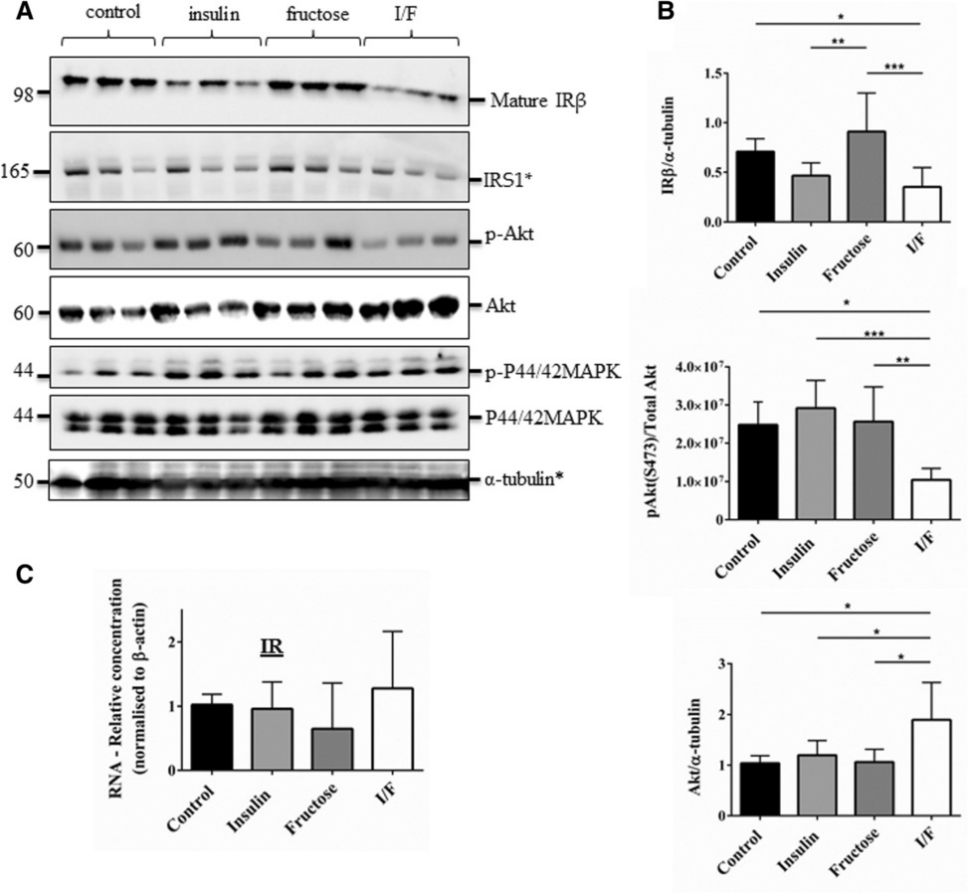
Impairment of insulin signalling using a combined insulin-fructose treatment protocol. Human astrocytes treated with ± 1 μM insulin ± 1 mM fructose for 4d (**a**). Representative immunoblots showing reductions in IRβ and pAkt with no impact on total IRS1 or downstream signalling through p44/42 MAPK. *α-tubulin was used as a loading control for blots and a representative loading control is shown. Molecular weight markers are indicated (kDa). **b** Bar charts show quantification of immunoblots, pAkt was normalised to Akt/α-tubulin. **c** qPCR analysis of IRβ RNA at 4d shows no differences at the RNA level indicating receptor decreases are mediated by receptor degradation. Data are mean + SEM (*n* = 3, 3 replicates/experiments, One way ANOVA with post-hoc analysis, *p < 0.05, **p < 0.01, ***p < 0.001

To achieve a physiologically relevant approach to impair insulin signalling, we modified a combined insulin + fructose (I/F) treatment protocol, which has previously being used to induce insulin resistance in Chang liver cells [30]. Human astrocytes were treated with 1 μM insulin and/or 1 mM fructose and the impact on the insulin signalling pathway assessed. There were significant differences between groups for IRβ (One-way ANOVA; *p* = 0.0001), pAkt (*p* = 0.0008) and Akt (*p* = 0.0061) (Fig. 6a, b). Addition of insulin alone resulted in a non-significant reduction in IRβ (*p* = 0.1776 compared to control) and no change in Akt phosphorylation (pAkt S473) (Fig. 6a, b). Addition of fructose alone had no impact on either IRβ or pAkt S473 compared to control. When insulin and fructose (I/F) were added in combination for 4 days there was a significant reduction in mature IRβ (*p* = 0.0215 compared to control) and in pAkt S473 (*p* = 0.0199 compared to control). In contrast total Akt levels were significantly increased in response to treatment with I/F (*p* = 0.0105 compared to control) (Fig. 6a, b). There was no effect of I/F on total levels of the adaptor protein IRS1. To further investigate the effects of I/F on signalling, we examined p44/42 MAPK, another downstream target of the insulin/IGF1 signalling pathway [31, 32], and demonstrated there was no change in either the phosphorylation of p44/42 MAPK at Threonine 202/Tyrosine 204 (required for activation) or total p44/42 MAPK (Fig. 6a). We also assessed signalling at the later time-point of 7 days and found that although there was still a significant reduction in IRβ (*p* < 0.0001) there was no longer an impact on Akt activation (Additional file 4: Figure S4). To determine whether the changes in IR levels were a result of degradation of the protein or due to reduction of the mRNA, qPCR was performed and demonstrated no change in IR mRNA expression levels indicating changes in signalling are due to changes in the protein, likely to be a result of receptor degradation/internalisation (Fig. 6c).

We additionally assessed the impact of I/F on astrocytes derived from temporal resections, with a similar effect observed; pAkt was significantly reduced after treatment with I/F (*p* = 0.017 compared to control) and Akt significantly increased (*p* = 0.0057 compared to control) (Additional file 5: Figure S5) although the effect of I/F on IRβ was less clear.

### Impairing IGF1 signalling

**Fig. 7:**
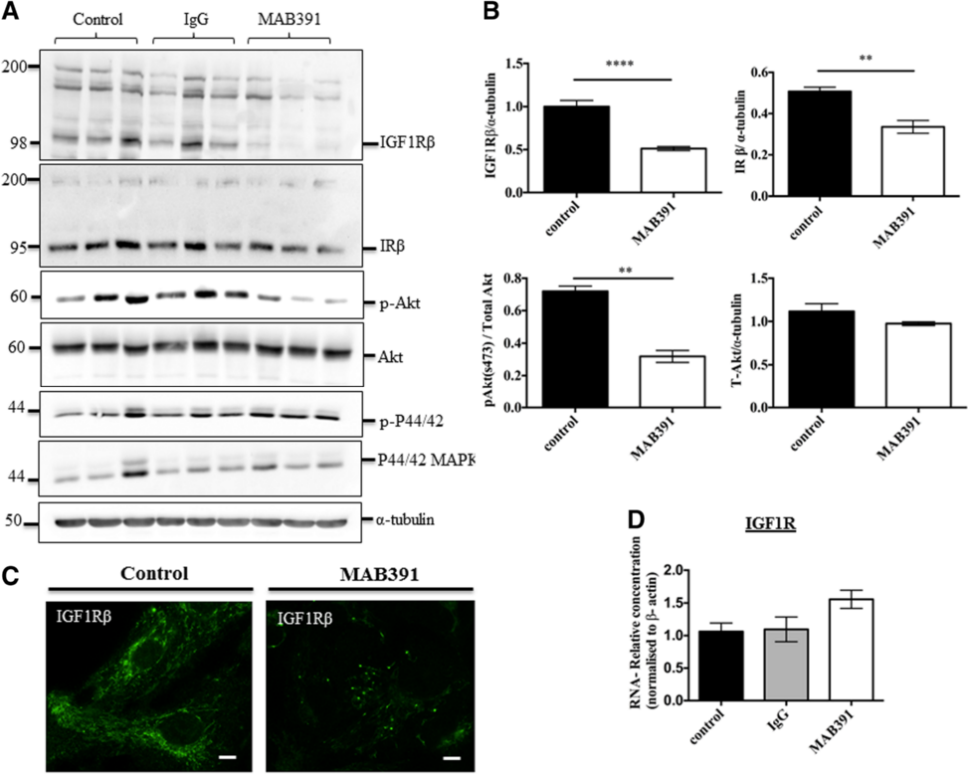
Impairment of IGF1 signalling using a monoclonal IGF1 antibody (MAB391). Human astrocytes treated with 11 μg/ml MAB391 for 24 h (**a**). Representative immunoblots demonstrate reductions in IGF1Rβ, IRβ and pAkt in response to MAB391 with no impact on total IRS1 or downstream signalling through p44/42 MAPK. *α-tubulin was used as a loading control for blots and a representative loading control is shown. Molecular weight markers are indicated (kDa). **b** Bar charts show quantification of immunoblots, pAkt was normalised to Akt/α-tubulin. **c** Immunofluorescence showing the reduction in IGF1R, scale bars represent 10 μM. **d** qPCR analysis of IGF1Rβ RNA after 24 h show no differences at the RNA level. Data represents mean + SEM (*n* = 3, 3 replicates/experiments, Unpaired *t*-test, **p < 0.01)

IGF1R levels were significantly reduced by treating astrocytes with 11 ug/ml of the IGF1R monoclonal antibody (MAB391) for 24 h as shown by both immunoblot analysis and immunofluorescence (Fig. 7a-c, unpaired *t*-test *p* = 0.0019). There was no effect of IgG control on IGF1R receptor levels or downstream signalling (Fig. 7a). Downstream signalling through Akt was significantly impaired as a result of IGF1R loss, as indicated by a decrease in pAkt S473 relative to untreated control (Fig. 7a-b, unpaired *t*-test *p* = 0.0012), but no changes in phospho p44/42 MAPK were observed (Fig. 7a). To identify whether MAB391 induced IGF1R receptor degradation or altered gene expression of the receptor qPCR analysis was performed. There was no difference in IGF1R mRNA between control and MAB391 groups (Fig. 7d, *p* = 0.111) indicating the decrease in receptor is at the protein level, likely due to internalisation and subsequent degradation of the receptor.

Treatment of astrocytes derived from temporal lobe resections with MAB391 effected a similar decrease in IGF1R (*p* < 0.0001), however there was no decrease in pAkt (Additional file 6: Figure S6).

### No effect of impaired insulin and IGF1 signalling on astrocyte growth/viability

The IIS pathway is involved in a number of key cellular functions including cell growth and regulation of the cell cycle. Therefore we investigated the impact of impaired insulin signalling on cell growth/viability using MTT and Cyquant assays. There was no change in cell viability as measured by MTT assay, and fluorimetric Cyquant® assays also showed no change in the total cell number (Additional file 7: Figure S7a). IGF1R depleted astrocytes were also subjected to Cyquant and MTT assays to assess cell growth/viability. Although there was a significant difference between control and MAB391-treated astrocytes this can be attributed to the addition of IgG to the cultures and not specifically to a reduction in IGF1R as addition of IgG induced a similar decrease (Additional file 7: Figure S7b). No changes were observed between control, IgG and MAB391 treated astrocytes, as shown by Cyquant analysis (Additional file 7: Figure S7b). We also determined the impact of reduced signalling on GFAP as a marker of astrocyte activation/reactivity. There was no change in GFAP levels in response to the combined I/F treatment (Additional file 7: Figure S7c). Treatment with MAB391 induced a significant increase in GFAP immunoreactivity as measured by immunoblot (Additional file 7: Figure S7c) (*p* = 0.0077 compared to control). However, there was no significant difference in GFAP levels between IgG treated and MAB391 treated astrocytes, (IgG versus MAB391; *p* = 0.262) which means the increase cannot solely be attributed to the effect of the reduction in IGF1R.

## Discussion

IIS has a role in neuronal growth, survival and metabolic function and inhibition of this pathway is associated with reduced neuronal survival, which is manifested in part through mitochondrial dysfunction and activation of pro-death signalling cascades [33]. Several studies have now documented reductions in IIS at the earliest stages of AD indicating that this signalling pathway may be involved in the development and/or progression of the disease. Several mechanisms have been implicated, for example changes in the phosphorylation state of IRS1, calpain overactivation and binding of Aβ-oligomers to IR [34–36]. To date, the focus of many studies has been on the impact of reduced IIS on neurons but astrocytes are also affected; a microarray analysis of the astrocyte transcriptome found the IIS pathway to be altered with increasing Braak stage, with downregulation of gene expression [21]. We demonstrate here that IIS is present and functional in cultured human astrocytes and that localisation of the adaptor protein, IRS1, is dependent on its phosphorylation state. We also describe two models by which insulin and IGF1 signalling can be selectively impaired by interventions that act at the levels of the respective receptors.

Insulin and IGF1 readily cross the blood brain barrier to exert effects within the central nervous system, and it has also been shown that insulin can be synthesised in the brain *de novo* by pyramidal neurons [37]. IR and IGF1 receptors are distributed widely throughout the brain, with IR highly abundant on neurons and IGF1R detected on both neurons and glia [5, 38, 39]. Here we show that both IR and IGF1R are present and functional in human primary astrocytes, through modulation of media components and immunoblotting for receptors and downstream signalling components. There are reported differences between peripheral and brain IR subunits; most brain IR subunits have a slightly lower molecular weight and are not downregulated in response to high insulin levels [40, 41]. However, Clarke et al. found that glial IR are downregulated in response to chronic insulin whereas neuronal IR are not [42] and it is now known that there are two isoforms of IR which are generated by alternative splicing; IR-A which excludes exon 11 and is expressed by neurons, and IR-B which includes exon 11, is expressed mainly in insulin-responsive tissues and in brain is predominantly expressed by glial cells [40]. We observed reductions in IR in response to insulin treatment, and showed that human astrocytes predominantly express the IR-B isoform of *Ir*. This finding contradicts those of Heni *et al*., [43] who found that IR-A was the predominant *Ir* isoform in human astrocytes. Although the regulation of *Ir* isoform expression is not well understood there are a number of potential reasons for this finding including the culturing conditions for the cells. We assessed *Ir* isoform levels in cells cultured in serum-containing media whereas Heni *et al*., starved their astrocytes for 48 h prior to a 15 min stimulation with insulin before assessing *Ir* isoform. In addition both studies used astrocytes derived from different sources, it is not known at what developmental stage the fetal-derived astrocytes used in our study or the Heni *et al*., study were isolated; since IR-A is important in development and is preferentially expressed in fetal cells [44] this could also explain the differing results. Our findings suggest that astrocytes and neurons might behave differently in response to an insulin resistant state and that the mechanism of impaired IIS may differ between these cell types.

The shift in molecular weight of the adaptor protein IRS1 in response to changes in serum, insulin or IGF1 supplementation is likely in part, due to phosphorylation events which modulate pathway activity although we cannot exclude the possibility that other post-translational modifications such as acetylation and O-GlcNAcylation are occuring. IRS1 can be phosphorylated at numerous serine/threonine phosphorylaton sites and tyrosine sites in order to regulate insulin/IGF1 signalling. Prolonged stimulation of RTKs results in the activation of numerous downstream kinases including c-Jun N-terminal kinase (JNK), protein kinase C (PKC) and glycogen synthase kinase (GSK) which, phosphorylate IRS1 at specific serine residues leading to downregulation of signalling [29].

Downstream signalling through Akt was modulated by the different component factors in the media and there were some slight differences in Akt signalling between the sources of astrocytes. This may be because the astrocytes are derived from different brain regions, that astrocytes derived from temporal lobe resections are dysfunctional [45] or because the commercially obtained astrocytes are fetal in origin whereas those from the temporal lobe are adult. It is, however, important to note that differentiated astrocytes from different sources vary in signalling responses [46].

Cellular localisation of the adaptor protein IRS1 was dependent on phosphorylation state. When phosphorylated at S616 and S636/639, we found that IRS1 localised to the nucleus, whereas IRS1 phosphorylated at tyrosine 612 (Y612) was present in both cytoplasmic and nuclear fractions. To our knowledge there are no previous studies specifically describing the nuclear localisation of serine phosphorylated IRS1, although a study by Reiss et al. describe a role for nuclear IRS1 in tumour development and progression [47]. These findings imply that IRS1 function in signalling is not confined to cytoplasmic cascades, but is more complex It is possible that since serine phosphorylation of IRS1 is typically associated with IIS inhibition, that this translocation event may represent IRS1 acting as a transcription factor, as is thought to occur with RTKs [22].

Immunohistochemistry for pIRS1 S616, S636/639 and Y612 on post mortem human tissue confirmed the *in vitro* findings, showing expression in neurons and small cells, some of which are likely to be glia. The more prominent nuclear localisation of serine phospho-forms was also seen in the post mortem tissue, showing that these subcellular localisations are of functional importance and not culture artefacts. These studies further indicated that there might be mislocalisation of IRS1 in neurons in AD, since there was reduced nuclear staining for serine phosphorylated IRS in neurons with NFTS and distinct labelling of structures that would be expected to be phospho-tau positive, namely NFTS, neuropil threads, neuritic plaques and GVD. Mislocalisation of TDP-43 is associated with inclusion formation in motor neuron disease [48]. So, in comparison, the staining of tau-pathological structures and nuclear exclusion might suggest a further mechanism for dysregulation of IIS in AD. This is an interesting question, but as the intention of the tissue work was to demonstrate *in vivo* localisation, its investigation is beyond the scope of the current study.

The IIS pathway, together with the downstream targets Akt and MAPK are downregulated in astrocytes as Alzheimer-type pathology develops [21]. IIS in the context of Alzheimer’s disease is currently the focus of much investigation with a number of studies investigating the potential of insulin supplementation as a therapeutic since it improves cognitive function [49–51]. However, there is still a considerable debate in the literature with regards to the exact roles of insulin and IGF1 in disease progression with studies reporting changes in both directions depending on the brain region and cell type studied [5, 34]. To begin to understand the functional implications of impaired signalling specifically in astrocytes we have developed models for impairing signalling through each of the cognate receptors.

The I/F model which has been described previously in Chang Liver cells [30], induces an insulin resistant state in these cells as measured by changes in glucose uptake and intracellular lipid accumulation. Therefore this model represents a relevant model for human ageing and neurodegeneration as diabetes is a risk factor for AD and might operate via this pathway. We have shown here that treatment of primary astrocytes with I/F results in IR degradation and an associated reduction in Akt activity (as measured by phosphorylation at S473). The reduction in IIS was observed after 4d; however, by 7d, although IR levels remained suppressed there was recovery in Akt signalling, indicating that other signalling pathways compensate for the reduction in signalling through the receptor. We also looked at signalling through p44/42 MAPK (ERK1/2) MAPK. Signalling through the Ras-Raf-MAPK pathway is involved in cell survival as well as cell cycle progression. There was no effect of I/F on the phosphorylated form of p44/42 MAPK. There could be multiple reasons for this, including that insulin signals preferentially through the PI3K-Akt signalling pathway or that the Ras-Raf-MAPK pathway is better preserved when insulin signalling is disrupted.

To impair IGF1 signalling we used a monoclonal IGF1R antibody to induce receptor degradation. It has previously been shown that long-term treatment with MAB391 induced receptor degradation and phosphorylation of the downstream target Akt in MCF7 human carcinoma cell [52]. Treatment with MAB391 similarly affected Akt and also had no effect on p44/42 MAPK as seen with I/F. We also observed reduction in the IR. This could be due to MAB391 binding directly to the IR, which may be possible due to the close homology of the IGF and insulin or because MAB391 is binding to receptor heterodimers formed between the IGF1R α-subunit and the IR β-subunit [53, 54]. Further, there was a differential effect between the 2 sources of astrocytes when treated with the IGF1R monoclonal antibody that was not seen with the I/F treatment. Astrocytes from temporal lobe resections did not show any change in Akt activity despite a clear reduction in IGF1R after treatment with MAB391. The reasons for this could be multi-fold, it could reflect differences between immature (fetal) and mature astrocytes (adult), or may be related to the differences seen in basal Akt activity between the two sources of astrocytes. It has previously been reported that cortical and midbrain astrocytes have differing dependencies on EGFR signalling [46] and therefore it could be speculated that differences in Akt activity may be present depending on the brain region from which astrocytes are derived. Our extensive characterisation of the astrocytes also demonstrated significant differences in astrocyte phenotype depending on the culturing media further highlighting the complexities of this cell type and signalling pathway.

Although the IIS pathway is critically involved in cell growth and proliferation we did not clearly demonstrate an effect of impaired insulin or IGFR signalling on these process. This might reflect the resilient nature of these cells, with functional redundancy between signalling pathways. Cross-pathway compensation between insulin and IGF1 signalling has been observed in cancer systems [55], so that downregulation of multiple pathways, as occurs in ageing brain, may be needed to significantly impair astrocyte survival. In cellular senescence, which governs ageing, there are multiple dysfunctional cellular processes which are controlled by networks of multiple signalling and feedback pathways further supporting the idea that the downregulation of multiple pathways is needed [56]. It is also possible that IIS reduction impairs the cells’ function in more subtle ways, for example, they may demonstrate an impaired stress response or may be less able to support other cells types; our future studies will be focused on understanding this. In support of this it has recently been shown that rodent astrocytes require IGF1 to protect neurons against oxidative injury [57].

IIS impairment in neurons has been suggested as a potential therapeutic target in AD and we now show that this system is also important in astrocytes. Astrocytes are a key component of the neurovascular unit, dysfunction of which is important in brain ageing and dementia [58]. How these changes affect this cell type is important in understanding the processes involved ageing and the development and progression of Alzheimer’s disease which is important in designing effective and targeted therapies that are able to restore function early on in the disease process.

## Methods

Unless otherwise stated all materials were obtained from Sigma Aldrich (Poole, Dorset, UK).

### Primary human astrocytes, LUHMES, and cell treatments

Human primary astrocytes were obtained from Sciencell Research Laboratories (Carlsbad, CA, US) and from temporal lobe resection during epilepsy surgical procedures [26]. Experiments were performed using commercially obtained Sciencell astrocytes with key experiments replicated in the additional source of astrocytes. Astrocytes were cultured in either Human Astrocyte media (Sciencell Research Laboratories) supplemented with fetal bovine serum (FBS), penicillin streptomycin and Astrocyte Growth Supplement (Sciencell Research Laboratories) or a 50:50 mix of F10:MEMα media (Gibco) supplemented with 10 % FBS and 1 % penicillin streptomycin. To characterise IIS, astrocytes were cultured in the presence and absence of 10 % FCS for 24 h and then supplemented with either 1 μM human recombinant insulin or 13.2 nM human recombinant IGF1 for 2 h. To impair signalling via the insulin receptor cultures were treated with either 1 μM insulin and/or 1 mM fructose (4d-7d). To impair signalling through IGF1R cultures were treated with 11 μg/ml IGF1 receptor monoclonal antibody (24 h) (MAB391, R&D Systems, MN, USA). This concentration is in line with that typically used [52] and correlates with the known EC50 for the antibody (11 μg/ml, R&D systems). Astrocytes were treated with MAB391 for 24 h prior to analysis of signalling pathways and an IgG control was included. Lund human mesencephalic (LUHMES) cells are conditionally-immortalised neuronal precursor cells which can be differentiated into post-mitotic neurons [59]. LUHMES were cultured on cell cultured flasks precoated with 50 μg/ml poly-l-ornithine (PLO) and 1 μg/ml fibronectin. Cells were grown in proliferation medium consisting of DMEM/F12 GlutaMAX™ supplement medium (Gibco), N2 supplement (Gibco) and 40 ng/ml recombinant basic fibroblast growth factor (Peprotech, NJ, U.SA). Once cells were 40–50 % confluent they were differentiated in differentiation media consisting of DMEM/F12 GlutaMAX™ supplement medium, N2 supplement and 1 μg/ml tetracycline. Two days after differentiation cells were trypsinised, counted and seeded at a density of 150,000 cells/cm^2^ on PLO-fibronectin coated plates and differentiated for a further 3 days prior to harvesting.

### Preparation of astrocyte lysates

After treatments, the medium was removed and cells were washed in ice-cold phosphate buffered saline (PBS), followed by lysis in extra strong lysis buffer (100 mM Tris–HCl (pH 7.5), 0.5 % (*w/v*) sodium dodecyl sulphate (SDS), 0.5 % (*w/v*) sodium deoxycholate, 1 % (*v/v*) Triton X-100, 75 mM sodium chloride (NaCl), 10 mM ethylenediaminetetraacetic acid, 2 mM sodium orthovanadate, 1.25 mM sodium fluoride, protease inhibitor cocktail and PhosStop (both Roche, Basel, Switzerland). Lysates were then sonicated followed by centrifugation at 17 000 g_(av)_ for 30 min at 4 °C. The protein concentration of supernatants was measured using a BCA protein assay kit (ThermoFisher Scientific, Waltham, MA, USA) and samples were standardised to equal protein concentration before being analysed by SDS-PAGE.

In addition to harvesting whole cell lysates, astrocytes were fractionated to yield cytoplasmic and nuclear fractions to confirm the subcellular localisation of specific pathway components. Cells were washed and pelleted as above prior to incubation with hypertonic buffer (20 mM Tris–HCl, 10 mM NaCl, 3 mM magnesium chloride, 1 mM phenylmethanesufonylfluoride (PMSF), 1 mM dithiothreitol (DTT), protease inhibitor cocktail and PhosStop) for 15 min. A 10 % NP40 solution was then added prior to centrifugation at 13 000 g_(av)_ for 5 min at 4 °C. The resulting supernatant was retained (cytosolic fraction) and the pellet resuspended in 50 μl cell extraction buffer (Invitrogen, plus 1 mM PMSF, 1 mM DTT, protease inhibitor cocktail and PhosStop) and incubated on ice for 30 min with samples vortexed at 10 min intervals. Finally samples were centrifuged at 14 000 g_(av)_ for 30 min at 4 °C and the supernatant retained and stored at −20 °C until required.

### SDS-PAGE and Immunoblotting

In total, 20-40 *μ*g protein was separated on 8 % or 12 % (*w/v*) SDS-PAGE gels and electrophoretically transferred to nitrocellulose membrane (GE Healthcare, Little Chalfont, Bucks, UK). After blocking with West Ezier Blocking Buffer (GenDEPOT, Barker, TX, US) for 30 min, membranes were incubated with primary antibodies overnight at 4 °C, followed by horseradish peroxidase (HRP)-conjugated secondary antibodies (DAKO, Copenhagen, Denmark) and ECL (EZ-ECL Biological Industries, Israel). Detected proteins were visualised and quantified using a G:Box Chemi-XT CCD Gel imaging system (Syngene, Cambridge, UK). The following primary antibodies were used for immunoblotting: insulin receptor beta (1/1000; rabbit monoclonal #3025, Cell Signalling Technology [CST], Beverly, MA, US), IGF1Rβ (1/200; Rabbit polyclonal sc-713, Santa Cruz BioTechnology [SCBT], Dallas, Texas, US), IRS1 (1/1000; Rabbit polyclonal #2382, CST), IRS2 (1/1000; Rabbit polyclonal #4502, CST), pIRS1 Y612 (1/1000; Rabbit polyclonal 44-816G, Life Technologies, Carlsbad, CA, US), pIRS1 S616 (1/1000; Rabbit polyclonal 44-550G, Life Technologies), pIRS1 S636/639 (1/1000; Rabbit polyclonal #2388, CST), Akt (1/1000; Rabbit polyclonal #4685, CST), pAkt S473 (1/2000; Rabbit monoclonal #4060, CST), p42-44 MAPK (1/1000; Rabbit polyclonal #9102, CST), phospho p42-44 MAPK (1/1000; Rabbit polyclonal #9101, CST), GFAP (1/1000, mouse monoclonal #3670, CST), γH2AX (1/1000, rabbit polyclonal AF228, R&D Systems), *β*-actin (1/5000; mouse IgG1 ab6276, clone AC-15; Abcam, Cambridge, UK) and α-tubulin (1/200, rabbit IgG ab18251, Abcam).

### Immunocytochemistry

Cultured cells were fixed in 4 % (*w/v*) paraformaldehyde in PBS for 5 min at 37 °C. Following fixation, cells were permeabilised (0.3 % (*v/v*) Triton X-100 in PBS) and blocked with 3 % (*w/v*) bovine serum albumin before incubation with polyclonal antibodies (1 h at ambient temperature) against IRS1 (1/50; Rabbit polyclonal sc7200, SCBT), pIRS1 S616 or pIRS S636/639 (both 1/100; CST,) and a monclonal antibody against vimentin (1/200; Rabbit polyclonal ab8978, Abcam). Cells were incubated with the appropriate species of secondary antibody for 1 h at ambient temperature (1/1000; Alexa-fluor conjugated, Life Technologies) and cell nuclei were stained with Hoescht 33342 (5 *μ*g/ml bisbenzimide in PBS). Astrocytes were examined using a Nikon DS-Ri1 Eclipse microscope (Nikon, Tokyo, Japan).

### RT-PCR for IR isoforms

Cultured astrocytes and LUHMES were washed with PBS and lysed in 110 μl (1 ml/10 cm^2^) Trizol (Life Technologies). RNA was isolated using Clean and Concentrator Columns (Zymo, Irvine, CA, US) and total RNA (approximately 500 ng) was incubated at 65 °C for 5 min and reverse transcribed at 42 °C for 50 min in a reaction mix containing qScript (Quanta Biosciences, Gaithersburg, MD, US). IR-A and IR-B were examined using primers 5′ and 3′ to exon 11 (F: GAATGCTGCTCCTGTCCAAA; R: TCGTGGGCACGCTGGTCGAG) and PCR performed using a G-storm Thermal Cycler (G-storm, Somerset, UK) with the following reaction profile: 94 °C for 30 s, 67 °C for 1 min, 72 °C for 30s for 35 cycles. Fragments 214 bp (IR-A) and 250 bp (IR-B) were resolved on 2.5 % agarose gels.

### qPCR

Astrocytes were harvested in Trizol as described above, RNA was isolated using Clean and Concentrator Columns and total RNA (approximately 500 ng) was incubated at 65 °C as above in a reaction mix containing qScript (Quanta Biosciences, Gaithersburg, MD, US). Primers for IR and IGF1R were designed, where possible, to span between adjacent exons (IR F: GCAGGAGCGTCATCAGCATA, R: TAACCCTAAACTTCCACCCACTGT; IGF1R F: ACCTCAACGCCAATAAGTTCGT, R:CGTCATACCAAAATCTCCGATTT). PCR amplification was performed using a Thermal Cycler (BioRad, Hercules, CA, USA) with the following reaction profile: 95 °C for 10 min, 95 °C for 30 s, 60 °C for 30 s for 40 cycles. The reaction mixture (20 μL) included 50 ng template cDNA, 10 μL SYBR green (Qiagen, Limburg, Netherlands) and 300 nM of each primer.

### MTT and Cyquant assays

The MTT (3-[4,5-dimethylthiazol-2-yl]-2,5 diphenyl tetrazolium bromide) assay is based on the conversion of MTT into formazan crystals by living cells, which determines mitochondrial activity and is widely used as a measure of cell viability. MTT (0.5 mg/mL) solution (110 μL) was added to each well of a 12 well plate, and the plates were incubated at 37 °C with 5 % CO_2_ for 3 h. Afterwards, 1.1 ml of 20 % sodium dodecyl sulphate (SDS) in 50 % dimethylformamide was added to each well, and the plates were incubated on a mini orbital shaker SSM1 at 150 rpm (Bibby Scientific, Stone, UK) for 3 h until formazan crystals were fully dissolved. The optical density of samples was then determined by measuring on a plate reader at 570 nm. In addition Cyquant NF assays (Life Technologies) were performed as an additional measure of cell number, assays were performed in accordance to the manufacturers instructions.

### Immunohistochemistry

*Post mortem* control (*n* = 3) and Alzheimer’s (*n* = 3) lateral temporal cortex (Brodmann area 21/22) and hippocampus (Table 1), were obtained from the Sheffield Brain Tissue Bank in accordance with Research Ethics committee approval (08/MRE00/103). Immunohistochemistry was performed on formalin fixed paraffin embedded tissue sectioned at 9 μM for pIRS1 S616 (Life Technology), pIRS1 S636/639 (CST) and pIRS1 Y612 (Merck Millipore, Rabbit polyclonal 09–432, Billerica, MA, USA) (antibody dilutions are detailed in Table 2). Sections were dewaxed, rehydrated, and endogenous peroxidase activity blocked with 3 % hydrogen peroxide in methanol. Sections were then subjected to antigen retrieval (see Table 2), blocked with 1.5 % normal serum for 30 min followed by incubation with primary antibody for 1 h at ambient temperature. To visualize antibody binding the HRP-conjugated avidin–biotin complex (Vectastain Elite kit, Vector Laboratories, Peterborough, UK) was used with diaminobenzidine (Vector Laboratories) as substrate. For dual staining experiments sections were subsequently blocked with 1.5 % normal serum, followed by an avidin-biotin block (Vector Laboratories) and incubation with GFAP primary antibody (DAKO, Rabbit polyclonal Z0334, Ely, UK). Sections were then incubated in 0.5 % biotinylated secondary antibody for 30 min followed by incubation with Alexa fluor®-488 streptavidin (Life Technologies) for 1 h at ambient temperature. Isotype-specific antibody controls confirmed specificity of staining. Images were captured using a Nikon DS Ri1 Eclipse.

**Table 1:** Age, sex and post mortem delay of cases

Case	Sex	Age	PMD
Control	M	63	Not recorded
Control	F	59	5 h
Control	M	67	63 h
Alzheimer’s disease	F	79	24 h
Alzheimer’s disease	F	71	Not recorded
Alzheimer’s disease	F	79	24 h

**Table 2:** Immunohistochemistry antibody source and specificity

Antibody	Species, dilution, incubation	Antigen retrieval
IRS1	Rabbit IgG, 1/25, 1 h RT	TSC, pH 6.5, MW 10 min
pIRS1 S616	Rabbit IgG, 1/100, 1 h RT	TSC, pH 6.5, MW 10 min
pIRS1 S636/639	Rabbit IgG, 1/50, 1 h RT	TSC, pH 6.5, MW 10 min
pIRS1 Y612	Rabbit IgG, 1/100, 1 h RT	EDTA, pH 8.0, MW 10 min
GFAP	Rabbit IgG, 1/500, ON, 4 °C	Dependent on initial probe (above)

*TSC* tris-sodium citrate, *EDTA* ethylenediaminetetraacetic acid, *RT* room temperature, *ON* overnight, *MW* microwave

### Statistics

Data were analysed using either Student’s unpaired *t*-test or one-way analysis of variance with Tukey’s post-hoc analysis (Graphpad Prism 5.0. Software, Graphpad Software Inc., La Jolla, CA, USA), differences were considered statistically significant when *P* < 0.05. Post hoc analyses were corrected for multiple comparisons and the multiplicity adjusted p-value is given.

## Additional files

Additional file 1: Figure S1. Immunofluorescence demonstrating expression of astrocyte markers in Sciencell astrocytes (A) lower magnification image showing the differing morphologies of the cultured astrocytes [labelled with vimentin] (B) Vimentin, (C) GFAP, (D) CD44. Scale bar represents 50 μM. (TIFF 3839 kb) Additional file 2: Figure S2. Effect of culturing media on human astrocytes. (A) Significant differences in cell growth and (B) expression of IRβ were observed depending on the media in which the cells were cultured in the. Cell growth measured by MTT assay, *n* = 3 (3 triplicates/experiment, unpaired *t* test * *p* < 0.05, **** *p* < 0.0001). Molecular weight markers are indicated (kDa) (TIFF 1723 kb) Additional file 3: Figure S3. The insulin/IGF1 signalling pathway was also characterised in astrocytes derived from temporal lobe resections. Astrocytes were cultured in either serum containing or serum deprived media with additional supplementation from recombinant human 1 μM insulin for 2 h. Immunoblots were probed with antibodies against IRβ, IGF1Rβ, IRS1, pAkt and total Akt. *β-actin was used as a loading control for blots and a representative loading control is shown. Molecular weight markers are indicated (kDa). (TIFF 2309 kb) Additional file 4: Figure S4. Impairment of insulin signalling using a combined insulin-fructose treatment protocol over a longer time-course. Human astrocytes treated with ± 1 μM insulin ± 1 mM fructose for 7d. (A) Immunoblots demonstrate clear reductions in IRβ but there is no reduction in pAkt. α-tubulin was used as a loading control for blots. Molecular weight markers are indicated (kDa). (B) Bar charts show quantification of immunoblots, pAkt was normalised to Akt/tubulin. Data represents mean + SEM (*n* = 3, 3 replicates/experiments, students unpaired *t*-test, *****p* < 0.0001). (TIFF 1772 kb) Additional file 5: Figure S5. Impairment of insulin signalling in astrocytes derived from temporal lobe resections using a combined insulin-fructose treatment protocol. Human astrocytes treated with ± 1 μM insulin ± 1 mM fructose for 4d. (A) Immunoblots for IRβ, pAkt, total Akt, pP44/42 MAPK and total p44/42 MAPK. β-actin was used as a loading control for blots. Molecular weight markers are indicated (kDa). (B) Bar charts show quantification of immunoblots, pAkt was normalised to Akt/tubulin. One way ANOVA with post hoc analysis, **p* < 0.05, ***p* < 0.01. (TIFF 2851 kb) Additional file 6: Figure S6. Reduced IGF1R but not downstream signalling in human astrocytes derived from temporal lobe resections using MAB391. Human astrocytes treated with MAB391 for 24 h. A) Immunoblots for IGF1R, pAkt, total Akt, pP44/42 MAPK and total p44/42 MAPK. α-tubulin was used as a loading control for blots. Molecular weight markers are indicated (kDa). (B) Bar charts showing quantification of blots, pAkt was normalised to Akt/tubulin. students unpaired *t*-test, **** *p* < 0.0001. (TIFF 1949 kb) Additional file 7: Figure S7. Functional impact of impaired insulin signalling. (A) Human astrocytes treated with ± 1 μM insulin ± 1 mM fructose were analysed by MTT assays and Cyquant assay at 4d. (B) Human astrocytes treated with MAB391 were analysed by MTT assays and Cyquant assays. (C) Treated astrocytes were also immunoblotted for GFAP immunoreactivity with α-tubulin as a loading control, representative immunoblot shown, bar chart shows quantification of immunoblots. Data represents mean ± SEM (*n* = 3, at least 3 replicates/experiment, one way ANOVA with post-hoc analysis, ***p* < 0.001). Molecular weight markers are indicated (kDa). (TIFF 7760 kb)

## Abbreviations

AD: Alzheimer’s disease
DTT: Dithiothreitol
EGFR: Epidermal growth factor receptor
FBS: Fetal bovine serum
GFAP: Glial fibrillary acidic protein
GSK: Glycogen synthase kinase
GVD: Granulovacular degeneration
HRP: Horseradish peroxidase
I/F: Insulin + fructose
IGF1: Insulin-like growth factor 1
IGF1R: Insulin-like growth factor receptor
IR: Insulin receptor
IRS: Insulin receptor substrate
JNK: c-Jun N-terminal kinase
LUHMES: Lund human mesencephalic cells
MAPK: Mitogen-activated protein kinase
NaCl: Sodium chloride
NFTs: Neurofibillary tangles
PCR: Polymerase chain reaction
PI3K: Phosphatidylinositol-4,5-bisphosphate 3-kinase
PKC: Protein kinase C
PMSF: Phenylmethanesufonylfluoride
RTK: Receptor tyrosine kinase
SDS: Sodium dodecyl sulphate
T2D: Type-2 diabetes
