# Supplementary figures and images for: Insulin and IGF1 signalling pathways in human astrocytes in vitro and in vivo; characterisation, subcellular localisation and modulation of the receptors
Source: Mol Brain. 2015 Aug 22;8:51. doi: 10.1186/s13041-015-0138-6 (PMC4546315; doi:10.1186/s13041-015-0138-6)

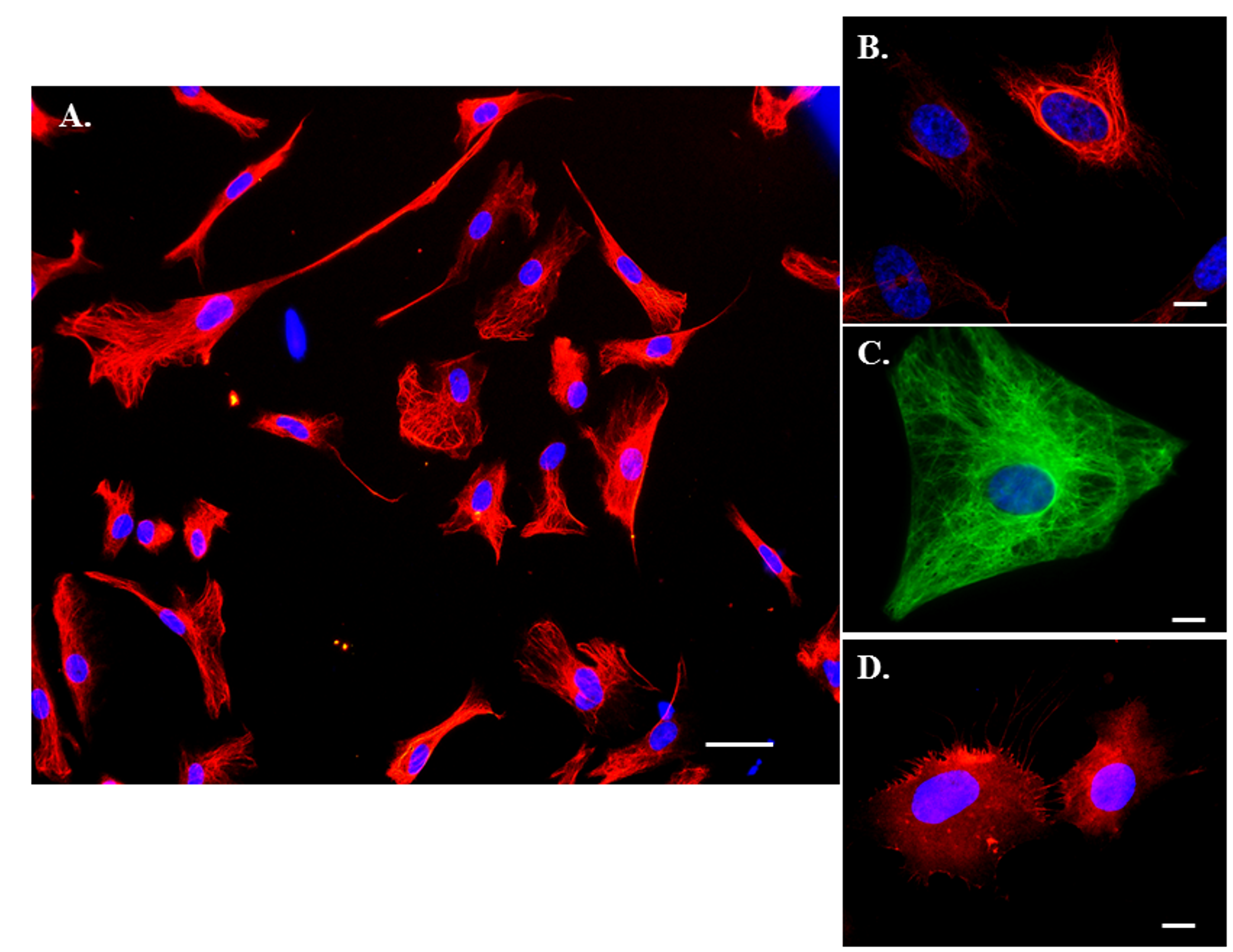

Supplement: Additional file 1: Figure S1. — Immunofluorescence demonstrating expression of astrocyte markers in Sciencell astrocytes (A) lower magnification image showing the differing morphologies of the cultured astrocytes [labelled with vimentin] (B) Vimentin, (C) GFAP, (D) CD44. Scale bar represents 50 μM. (TIFF 3839 kb) [file 13041_2015_138_MOESM1_ESM.tif]
